# Stress-related reduction of hippocampal subfield volumes in major depressive disorder: A 7-Tesla study

**DOI:** 10.3389/fpsyt.2023.1060770

**Published:** 2023-02-02

**Authors:** Judy Alper, Rui Feng, Gaurav Verma, Sarah Rutter, Kuang-han Huang, Long Xie, Paul Yushkevich, Yael Jacob, Stephanie Brown, Marin Kautz, Molly Schneider, Hung-Mo Lin, Lazar Fleysher, Bradley N. Delman, Patrick R. Hof, James W. Murrough, Priti Balchandani

**Affiliations:** ^1^Department of Diagnostic, Molecular, and Interventional Radiology, Icahn School of Medicine at Mount Sinai, New York, NY, United States; ^2^Department of Biomedical Engineering, City College of New York, New York, NY, United States; ^3^Department of Neurosurgery, Icahn School of Medicine at Mount Sinai, New York, NY, United States; ^4^Department of Psychiatry, Icahn School of Medicine at Mount Sinai, New York, NY, United States; ^5^Department of Radiology, University of Pennsylvania, Philadelphia, PA, United States; ^6^Population Health Science and Policy Department, Icahn School of Medicine at Mount Sinai, New York, NY, United States; ^7^Nash Family Department of Neuroscience and Friedman Brain Institute, Icahn School of Medicine at Mount Sinai, New York, NY, United States

**Keywords:** depression, hippocampus, magnetic resonance imaging, stress, subfields, ultrahigh field MRI

## Abstract

**Background:**

Major depressive disorder (MDD) is a prevalent health problem with complex pathophysiology that is not clearly understood. Prior work has implicated the hippocampus in MDD, but how hippocampal subfields influence or are affected by MDD requires further characterization with high-resolution data. This will help ascertain the accuracy and reproducibility of previous subfield findings in depression as well as correlate subfield volumes with MDD symptom scores. The objective of this study was to assess volumetric differences in hippocampal subfields between MDD patients globally and healthy controls (HC) as well as between a subset of treatment-resistant depression (TRD) patients and HC using automatic segmentation of hippocampal subfields (ASHS) software and ultra-high field MRI.

**Methods:**

Thirty-five MDD patients and 28 HC underwent imaging using 7-Tesla MRI. ASHS software was applied to the imaging data to perform automated hippocampal segmentation and provide volumetrics for analysis. An exploratory analysis was also performed on associations between symptom scores for diagnostic testing and hippocampal subfield volumes.

**Results:**

Compared to HC, MDD and TRD patients showed reduced right-hemisphere CA2/3 subfield volume (*p* = 0.01, *η*^2^ = 0.31 and *p* = 0.3, *η*^2^ = 0.44, respectively). Additionally, negative associations were found between subfield volumes and life-stressor checklist scores, including left CA1 (*p* = 0.041, *f*^2^ = 0.419), left CA4/DG (*p* = 0.010, *f*^2^ = 0.584), right subiculum total (*p* = 0.038, *f*^2^ = 0.354), left hippocampus total (*p* = 0.015, *f*^2^ = 0.134), and right hippocampus total (*p* = 0.034, *f*^2^ = 0.110). Caution should be exercised in interpreting these results due to the small sample size and low power.

**Conclusion:**

Determining biomarkers for MDD and TRD pathophysiology through segmentation on high-resolution MRI data and understanding the effects of stress on these regions can enable better assessment of biological response to treatment selection and may elucidate the underlying mechanisms of depression.

## 1. Introduction

Major depressive disorder (MDD) is a debilitating illness of high prevalence worldwide (1) that affects ~6.9% of the U.S. population annually. Current treatments for MDD are lacking due to limited treatment efficacy and significant lag time to onset of therapeutic benefit. Although several mechanisms for depression have been proposed in the literature (2–6), the pathophysiology underlying psychological dysfunction that characterizes the disease is still not clearly understood. Determining measurable neurobiological biomarkers for pathology in MDD may enable better assessment of biological response to treatment selection and timing, highly specific criteria for diagnosis by which to differentiate disease subtypes, and development of novel treatments targeting disease mechanisms. Elucidating the complex interactions between brain biomarkers and clinical characteristics of MDD would allow for greater integration of anatomical biomarkers in clinical diagnoses and treatments.

In addition to understanding the biological basis for MDD, further analysis is warranted to understand treatment-resistant depression (TRD), as there are many patients for whom no sufficient treatment exists (7). It is estimated that 20%–30% of patients with depression experience resistance to treatment (8), and TRD represents approximately half of the treatment costs for MDD overall (9).

A major brain structure known to be implicated in neuropsychiatric diseases is the hippocampus. For perspective, previous magnetic resonance imaging (MRI) studies have shown that structural abnormalities in the hippocampus are often found in post-traumatic stress disorder (10), Alzheimer’s disease (11), and depression (12). Furthermore, meta-analyses of large datasets indicate hippocampal grey matter volume is commonly diminished in MDD (13, 14).

Anatomically, the hippocampus consists of cytoarchitecturally defined fields and includes Ammon’s horn (and its four subdivisions, CA1-4), the dentate gyrus (DG), and the subiculum (SUB) (15). A variety of neuropsychiatric conditions may involve the hippocampus overall or may differentially involve various hippocampal subfields, as these regions are morphologically and functionally different from one another (16).

Animal studies (17, 18) as well as post-mortem analyses (19, 20) have confirmed that hippocampal subfields are differentially affected by neuropsychiatric diseases (15, 16) and have further reinforced an association between hippocampal subfield volume changes and MDD in humans (21–23). It is possible that volumetric reductions in subfield volumes result from deleterious neurotoxic effects of stress-related hormones on neurons and glial cells (24). Therefore, hippocampal subfield volumes may serve as sensitive biomarkers for the disease. While many studies in depression have been performed, there is still a lack of consistency in the reported subfields with significant findings and validation of existing findings is warranted (25).

MRI has been used to inspect hippocampal subfield volumes *in vivo* (26–30) at varying field strengths. Use of ultra-high field MRI scanners, such as those operating at 7-Tesla (7T), can allow for increased visualization of boundaries and delineation of subfields than conventional clinical strength scanners due to superior contrast and resolution at 7T (31). These high-field advantages can reveal structural subtleties which are below the threshold of detectability at clinical field strengths (32). There is a limited number of 7T studies examining hippocampal subfields in MDD and their segmentation methods include either manual tracings (33) or automated segmentation with FreeSurfer version 6.0 (29, 34, 35). Manual hippocampal subfield segmentation is labor-intensive and can be difficult to reproduce across research centers (36). The applicability of automated segmentation of hippocampal subfields with FreeSurfer 6.0 has been validated on field strengths lower than 7T (up to 3T) and the atlas used was built from manual tracings in a cohort of elderly subjects, potentially limiting its applicability in studies with younger populations (37). Additionally, some of the subfield boundaries, including the CA4/GC-DG interface and the interfaces between the CA fields along the pyramidal layer of the hippocampus, cannot be clearly visualized in the training data used for the atlas in FreeSurfer 6.0. Overall, there is an ongoing challenge in hippocampal subfield MRI literature, such that there is large discrepancy and variability in subregion definitions and boundaries (25). The fact that there may be variability in subfield delineations warrants further analyses employing other segmentation methods, such as the trained automatic segmentation of hippocampal subfields (ASHS) software (38), to verify and validate results in the MDD cohort.

In this study, we evaluated differences in hippocampal subfield volumes between MDD patients and healthy controls, as well as between a subset of TRD patients and healthy controls. We also performed an exploratory analysis on associations between symptom scores for diagnostic testing, including the Montgomery-Asberg Depression Rating Scale (MADRS) and Life Stressor Checklist (LSC), and hippocampal subfield volumes. These analyses were enabled by acquisition of high-resolution data using 7T MRI and applying ASHS software to perform automated hippocampal subfield segmentation. ASHS is unique in that it has been validated on ultra-high field MRI data (36, 37) and allows for user-defined segmentation protocols through its training pipeline. We used manual hippocampal subfield tracings to generate a specialized 7T atlas for ASHS training to enable increased segmentation accuracy based on our high-resolution 7T data. To our knowledge, this study is the first to apply ASHS to 7T MDD data.

Investigation of hippocampal subfield volumes at higher field strengths using segmentation methods optimized for ultra-high field data can shed light on the accuracy and reproducibility of previous subfield findings in depression as well as association of subfield volumes and MDD symptom scores. Determining biomarkers for pathology in MDD through segmentation on high-resolution MRI data can ultimately enable better assessment of biological response to treatment selection and timing as well as development of novel treatments targeting disease mechanisms.

## 2. Methods

### 2.1. Subjects

A total of 63 subjects were included in this study (39 biologic males, 23 biologic females, and one other) including 35 MDD patients (mean age 39.5 years, standard deviation 11.8 years, 20 males, 14 females, and one other) and 28 healthy controls (mean age 39.4 years, standard deviation 10.4 years, 19 males and 9 females). Age was not significantly different between groups (*p* = 0.99). All participants provided informed written consent prior to investigation. The protocol used was approved by the local Institutional Review Board, namely the Human Research Protection Program at the Icahn School of Medicine at Mount Sinai.

Prior to the MRI visit, a series of symptom questionnaires were administered to all participants by trained clinical raters. Included in these were the Structural Clinical Interview for DSM-IV Axis I Disorders (SCID) or the Structured Clinical Interview for DSM-5 Research Version (SCID-5), the Montgomery-Asberg Depression Rating Scale (MADRS; range 0–60; higher score indicates greater depression severity) and Life Stressor Checklist (LSC; range 0–30, higher score indicates greater number of exposures to stressful life events). Lifetime number of antidepressant failures were also collected. The demographic and clinical characteristics are presented in Table 1.

**Table 1:** Demographic and clinical characteristics.

	MDD (*n* = 35)	HC (*n* = 28)	Value of *p*
Male (frequency, %)	20, 57.14%	19, 67.86%	0.46
Age, years (mean ± SD)	39.46 ± 11.77	39.43 ± 10.35	0.99
Age at first episode (mean ± SD)	18.46 ± 14.07	–	–
Duration of current episode, months (mean ± SD)	47.52 ± 71.80	–	–
Number of antidepressant failures (mean ± SD)	1.25 ± 2.02 (32)	–	–
MADRS	22.94 ± 11.93	0.50 ± 1.07	2.58E-14*
LSC	5.15 ± 4.35 (26)	2.32 ± 2.13	0.0034*

MADRS, Montgomery-Asberg Depression Rating Scale; LSC, Life Stressor Checklist.

^*^Significant value of *p* < 0.05 for MDD group compared to HC group.

All subjects underwent MRI scanning at 7T (Magnetom, Siemens) under an approved protocol by the Institutional Review Board at the Icahn School of Medicine at Mount Sinai. To qualify for the study, MDD participants needed to have a current primary diagnosis of MDD based on clinical evaluation using the SCID or SCID-5. All MDD participants were antidepressant free for at least 4 weeks prior to study participation. Healthy control (HC) participants were also assessed using the SCID or SCID-5 and were excluded for any current or lifetime psychiatric conditions. Participants in both groups with a current diagnosis of obsessive–compulsive disorder, with alcohol or substance use disorder in the past year, or with a lifetime history of psychosis, neurological disease, or bipolar disorder were excluded. Participants with contraindications to 7T MRI were also excluded. TRD patients were defined as patients with a lifetime history of one or more anti-depressant failures. This is a non-standard definition for TRD and, therefore, results in this subset of patients would be considered very preliminary.

### 2.2. Image acquisition protocol

A 32-channel Nova Medical head coil was used to acquire brain images for segmentation. The 90-min imaging protocol included MP2RAGE (TR 6,000 ms, TI1 1,050 ms, TI2 3,000 ms, TE 5.06 ms, voxel 0.70 × 0.70 × 0.70 mm^3^) and T_2_ TSE (TR 9,000 ms, TE 69 ms, voxel 0.45 × 0.45 × 2 mm^3^) scans acquired at a coronal oblique oriented perpendicular to the long axis of the hippocampus.

### 2.3. Hippocampal subfield segmentation

Prior to performing automated hippocampal subfield segmentation manual subfield tracings were performed using 3DSlicer software on high-resolution 7T T_2_-TSE images (0.45 × 0.45 × 2 mm^3^). The tracing method was guided and verified by an expert neuroanatomist (PH) and neuroradiologist (BD) and the segmentations were generated through the joint effort of two trained image analysts, including the following subfields: CA1, CA2/3, CA4/DG, subiculum, and pre-subiculum.

As ASHS performs hippocampal subfield segmentation based on existing brain atlases, the developers of the ASHS platform allowed for atlas building, which can enable increased segmentation accuracy based on advantages of a given set of data. Therefore, we used the manual tracings described above to generate a specialized 7T atlas for ASHS training.

The atlas contained manually-traced hippocampi on 7T images on a combined subset of 15 MDD patients and controls with no clinically significant, identifiable hippocampal abnormalities. The manually segmented subsample consisted of 6 females, with a mean age of 41.3 years and 9 males, with a mean age of 48.1 years. A left–right flip of each segmentation was performed, resulting in a total of 30 subjects for ASHS atlas training. The atlas was approved upon visual inspection by an expert neuroradiologist (BD) and neuroanatomist (PH).

Automated hippocampal subfield segmentation using the specialized 7T atlas for ASHS was applied to 7T MRI data on MDD patients and healthy controls. The subfields delineated by ASHS are the same as those segmented in the manual tracings used for training and included CA1, CA2/3, CA4/DG, subiculum, and pre-subiculum. High resolution T_1_ and T_2_ weighted images were used as inputs to perform optimal segmentation with ASHS.

### 2.4. Statistics

All hippocampal subfields were normalized to total brain volume provided by FreeSurfer version 6.0. For this normalization, each subfield volume was divided by the total brain volume of a given subject and multiplied by a scaling factor. We elected to combine the subiculum and presubiculum into one region (subiculum total) for analysis in order to overcome inconsistencies between methods in segmenting these regions (25). Because the data were not normally distributed, the nonparametric Mann–Whitney *U*-test was used to compare subfield volumes by group. Effect sizes (*η*^2^) were also calculated. A proportional odds ordinal logistic regression model was also used to compare subfield volumes by group, controlling for age and biological sex. *β* values were also calculated. A multivariate ordinary least squares regression model was applied to predict symptom scores, specifically MADRS and LSC score, from hippocampal subfield volumes, controlling for age and biological sex. Effect sizes (*Cohen’s f* ^2^) were also calculated. Significance was set at an *α* level of 0.05 for all tests. Correction for multiple comparisons was performed on all findings using adaptive false discovery rate (FDR) (39).

## 3. Results

**Figure 1:**
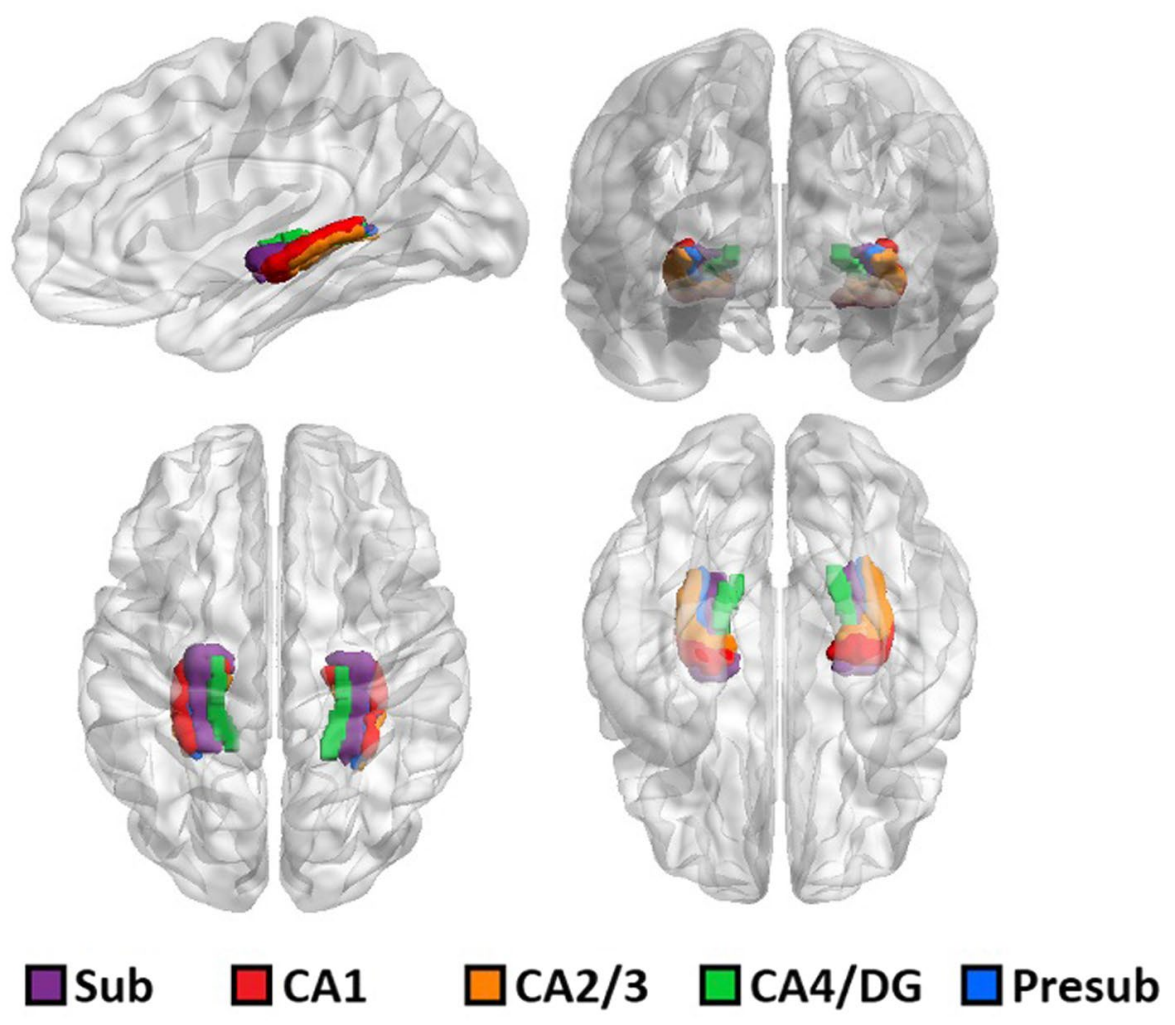
3D rendering of one representative subject’s brain with an overlay of the automated hippocampal subfields segmentation.

The results of ASHS hippocampal subfield segmentation for one representative subject are shown in Figure 1.

### 3.1. Group comparisons of subfield volumes

#### 3.1.1. Group comparison of subfield volumes in MDD patients and controls

**Figure 2:**
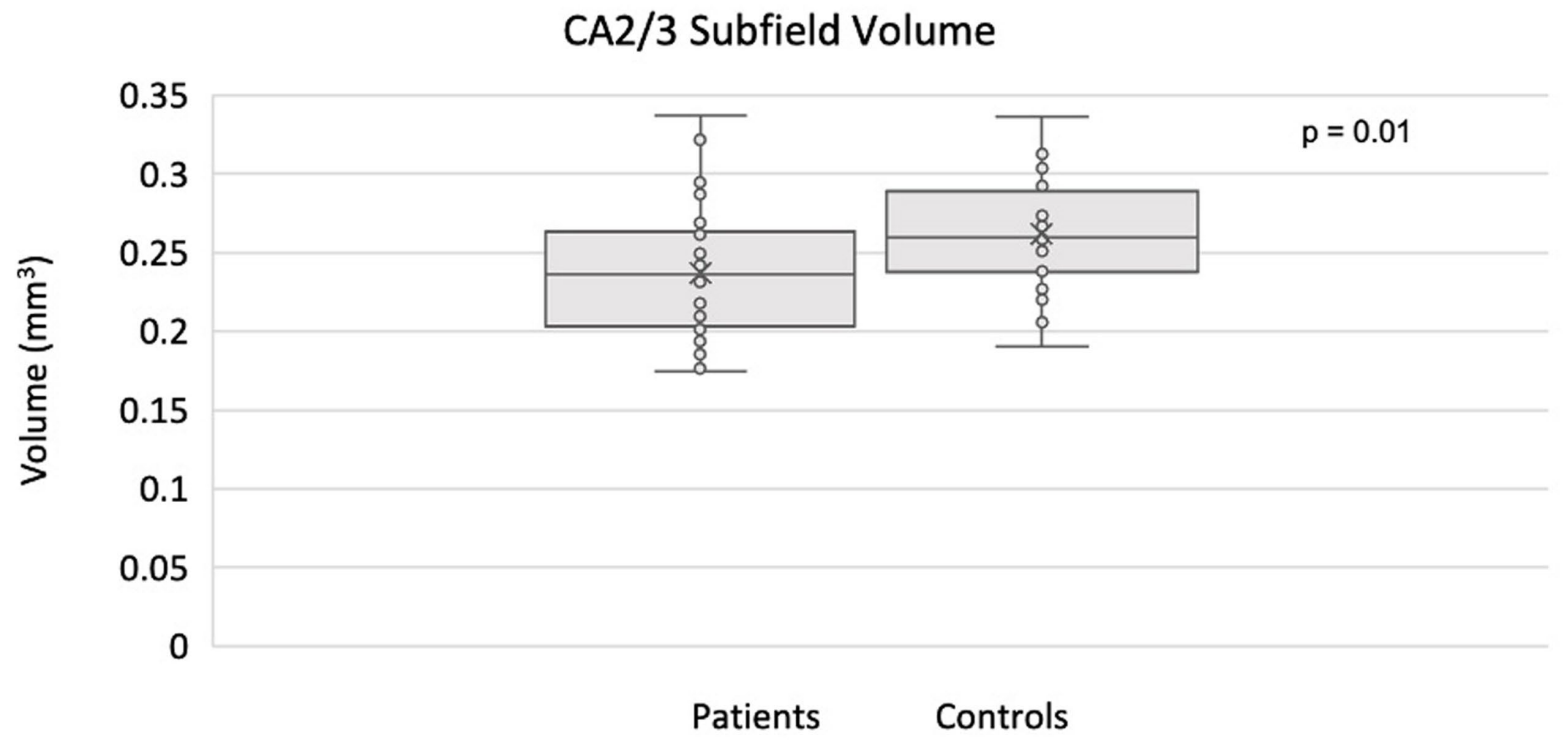
Smaller CA2/3 subfield volume in the right hippocampus of MDD patients compared to controls using ASHS.

The full set of results for this group comparison is shown in Tables 2A,B. Table 2A summarizes the results of the Mann–Whitney *U*-test and Table 2B summarizes the results of the proportional odds ordinal logistic regression model, controlling for age and biological sex. The volume of the CA2/3 subfield on the right side was found to be significantly smaller in patients compared to controls in Table 2A (*p* = 0.01, *η*^2^ = 0.31), as shown in Figure 2, and in Table 2B (*p* = 0.02, *β* = 1.12). No subfield was significant after correction for multiple comparisons using the adaptive FDR method.

**Table 2A:** Group differences in volumes between MDD patients and healthy controls.

Hippocampal subfield	Left hemisphere		Right hemisphere	
	*Raw*/*Adj p*	*η* ^2^	*Raw*/*Adj p*	*η* ^2^
CA1	0.62/0.79	0.06	0.75/0.75	0.04
CA2/3	0.08/0.38	0.22	0.01*/0.06	0.31
CA4/DG	0.69/0.79	0.05	0.16/0.36	0.18
Subiculum total	0.79/0.79	0.03	0.74/0.75	0.04
Hippocampus total	0.62/0.79	0.06	0.22/0.36	0.16

^*^Significant *p*-value < 0.05 in right hemisphere CA2/3 without correction for multiple comparisons. No subfield was significant after corrected for multiple comparisons by brain side using the adaptive false discovery method Mann–Whitney *U*-test; effect sizes *η*^2^ ≥ 0.1, *η*^2^ ≥ 0.3, and *η*^2^ ≥ 0.5 represent small, medium, and large effect sizes, respectively; (*n* = 63; 35 MDD, 28 HC).

**Table 2B:** Group effect in volumes between MDD patients and healthy controls, adjusting for age and sex using proportional odds ordinal logistic regression model.

Hippocampal subfield	Left hemisphere		Right hemisphere	
	*Raw*/*Adj p*	*β* (SE)	*Raw*/*Adj p*	*β* (SE)
CA1	0.67/0.93	0.19 (0.44)	0.54/0.66	0.27 (0.45)
CA2/3	0.06/0.32	0.84 (0.45)	0.02*/0.08	1.12 (0.46)
CA4/DG	0.80/0.93	0.11 (0.44)	0.15/0.27	0.65 (0.45)
Subiculum total	0.93/0.93	−0.04 (0.44)	0.66/0.66	0.20 (0.44)
Hippocampus total	0.57/0.93	0.25 (0.45)	0.16/0.27	0.63 (0.45)

^*^Significant *p*-value < 0.05 in right hemisphere CA2/3 without correction for multiple comparisons. No subfield was significant after corrected for multiple comparisons by brain side using the adaptive false discovery method; (*n* = 63; 35 MDD, 28 HC).

#### 3.1.2. Group comparison of subfield volumes in TRD subset and controls

**Figure 3:**
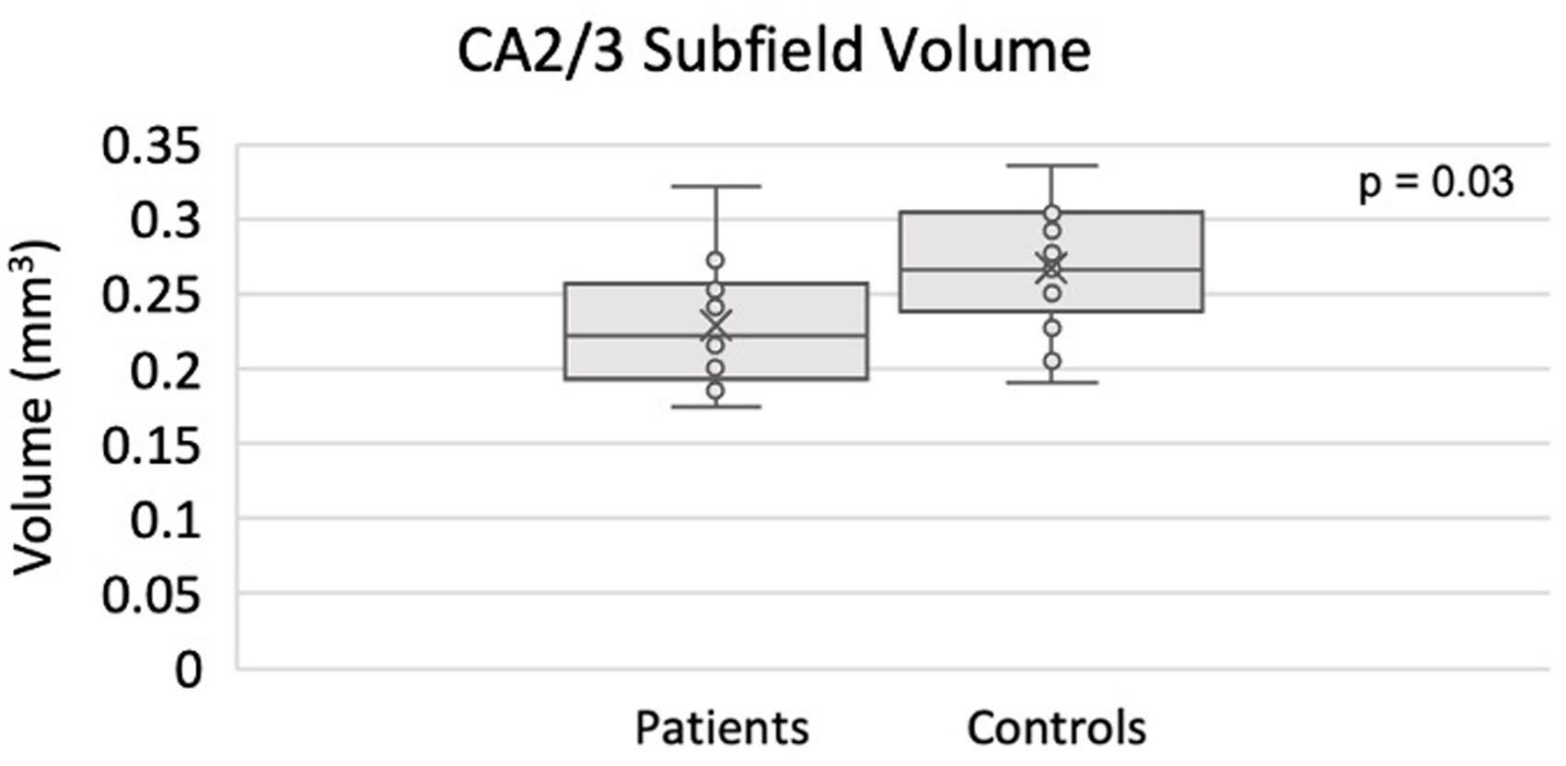
Smaller CA2/3 subfield volume in the right hippocampus of TRD patients compared to controls using ASHS.

The full set of results for this group comparison is shown in Tables 3A,B. Table 3A summarizes the results of the Mann–Whitney *U*-test on 13 TRD patients (eight males, five females) and 13 HC. Table 3B summarizes the results of the proportional odds ordinal logistic regression model, controlling for age and biological sex, on 13 TRD patients and 28 HC. The volume of the CA2/3 subfield on the right side was found to be significantly smaller in patients compared to controls in Table 3A (*p* = 0.03, *η*^2^ = 0.44), as shown in Figure 3, and in Table 3B (*p* = 0.01, *β* = −1.77). The right CA2/3 subfield volume finding, identified through the proportional odds ordinal logistic regression model, was significant after correction for multiple comparisons (*p* = 0.04) using the adaptive FDR method.

**Table 3A:** Group differences in volumes between TRD patients and healthy controls.

Hippocampal subfield	Left hemisphere		Right hemisphere	
	*Raw*/*Adj p*	*η* ^2^	*Raw*/*Adj p*	*η* ^2^
CA1	0.82/0.82	0.05	0.23/0.29	0.24
CA2/3	0.23/0.82	0.24	0.03*/0.13	0.44
CA4/DG	0.46/0.82	0.15	0.13/0.24	0.3
Subiculum total	0.78/0.82	0.06	0.82/0.82	0.05
Hippocampus total	0.59/0.82	0.11	015/0.24	0.29

^*^Significant *p*-value < 0.05 in right hemisphere CA2/3, corrected for multiple comparisons by brain side using the adaptive false discovery rate method, Mann–Whitney *U*-test; effect sizes *η*^2^ ≥ 0.1, *η*^2^ ≥ 0.3, and *η*^2^ ≥ 0.5 represent small, medium, and large effect sizes, respectively; (*n* = 26; 13 MDD, 13 HC).

**Table 3B:** Group effect in volumes between TRD patients and healthy controls, adjusting for age and sex using proportional odds ordinal logistic regression model.

Hippocampal subfield	Left hemisphere		Right hemisphere	
	*Raw*/*Adj p*	*β* (SE)	*Raw*/*Adj p*	*β* (SE)
CA1	0.86/0.86	−0.11 (0.61)	0.28/0.35	−0.67 (0.28)
CA2/3	0.12/0.60	−0.97 (0.63)	0.01*/0.04*	−1.77 (0.66)
CA4/DG	0.81/0.86	−0.14 (0.61)	0.10/0.23	−1.02 (0.63)
Subiculum total	0.56/0.86	0.36 (0.61)	0.77/0.77	0.18 (0.61)
Hippocampus total	0.73/0.86	−0.21 (0.61)	0.14/0.23	−0.93 (0.62)

^*^Significant *p*-value < 0.05 in right hemisphere CA2/3, corrected for multiple comparisons by brain side using the adaptive false discovery rate method; (*n* = 41; 13 MDD, 28 HC).

### 3.2. Regression analysis predicting symptom scores from subfield volumes

In a subset of 26 patients (14 males, 12 females) and 28 healthy controls (19 males, nine females) for which symptom scores were collected, linear regressions, with age and sex as covariates, were performed to assess the relationship between symptom scores and hippocampal subfield volumes.

**Figure 4:**
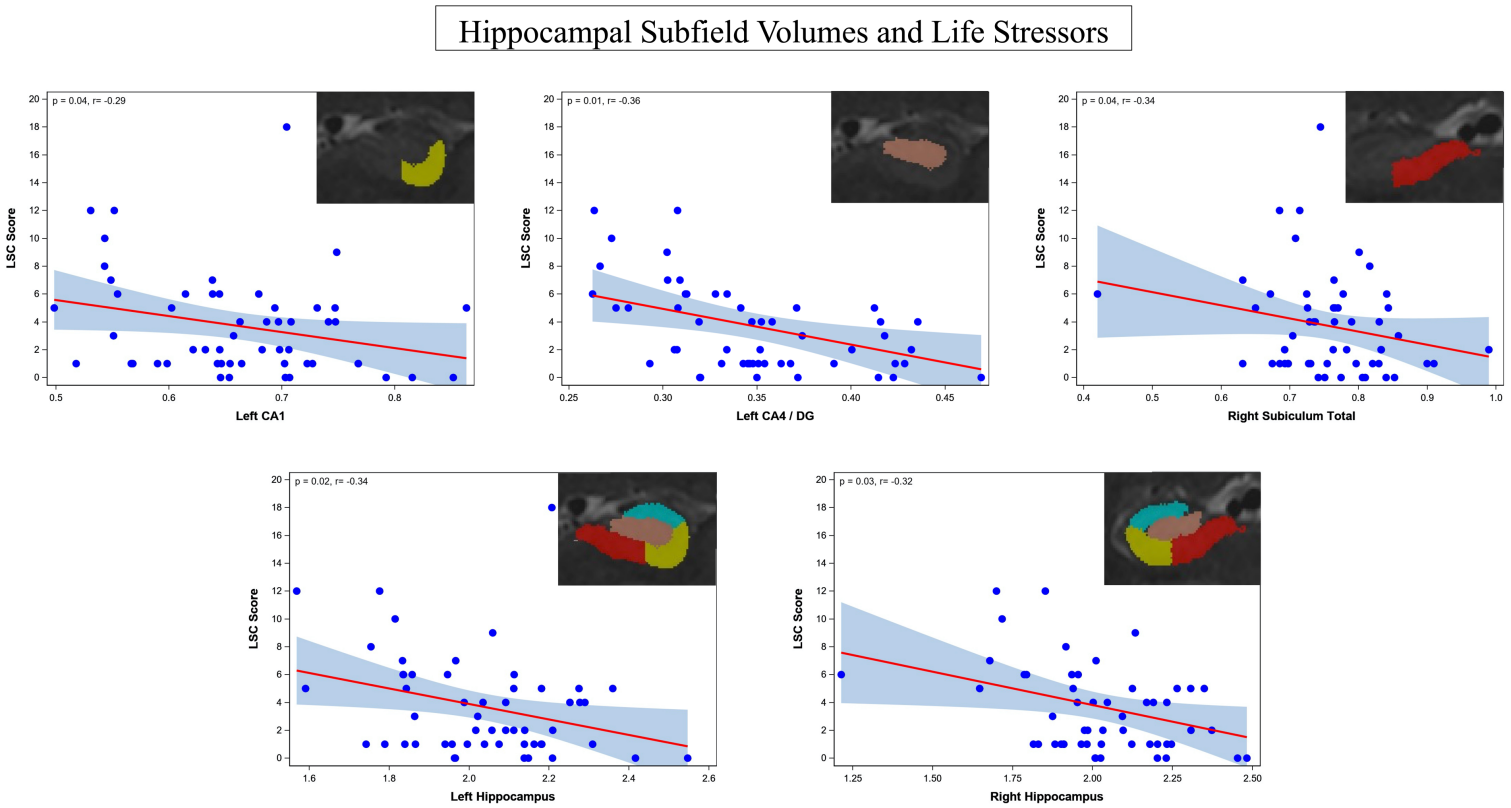
Regression plots of the identified significant negative associations between hippocampus subfield CA1, combined CA4/DG, total subiculum, left whole hippocampus, and right whole hippocampus and life stressors rated by the LSC.

The ASHS generated subfield volumes that had significance in association with LSC scores included left CA1 (*p* = 0.04, *f*  ^2^ = 0.419), left CA4/DG (*p* = 0.01, *f*  ^2^ = 0.584), and right subiculum total (*p* = 0.04, *f*  ^2^ = 0.354), all of which indicated a negative relationship between subfield volume and LSC score. Similarly, the left hippocampus total volume (*p* = 0.02, *f*  ^2^ = 0.134) and right hippocampus total volume (*p* = 0.03, *f*  ^2^ = 0.110) were also significant in their negative association with LSC scores. Regression plots with ASHS software significant findings are shown in Figure 4. All significant findings survived multiple comparison correction with adaptive FDR. The full set of results for the associations between LSC scores and hippocampal subfields are shown in Table 4. The MADRS score and other symptom scores collected showed no significant association with subfield volumes.

**Table 4:** Regression analysis findings for LSC score and hippocampal subfield volumes in MDD patients and healthy controls.

Hippocampal subfield	Left hemisphere		Right hemisphere	
	*Raw*/*Adj p*	Cohen’s *f*^2^	*Raw*/*Adj p*	Cohen’s *f*^2^
CA1	0.04*/0.04*	0.419	0.13/0.13	0.271
CA2/3	0.08/0.08	0.389	0.18/0.18	0.281
CA4/DG	0.01*/0.02*	0.584	0.14/0.14	0.696
Subiculum total	0.23/0.23	0.423	0.04*/0.04*	0.354
Hippocampus total	0.02*/0.02*	0.134	0.03*/0.04*	0.11

*Significant *p*-value < 0.05 in left hemisphere CA1 and CA4/DG, right hemisphere subiculum total, and hippocampus total in the left and right hemispheres, corrected for multiple comparisons by brain side using the adaptive false discovery method; local effect sizes *f* ^2^ ≥ 0.02, *f* ^2^ ≥ 0.15, and *f* ^2^ ≥ 0.35 represent small, medium, and large effect sizes, respectively; (*n* = 54; 26 MDD, 28 HC).

## 4. Discussion

In this study, we used high-resolution 7T MRI data to train the ASHS software for hippocampal subfield segmentation and analyze hippocampal subfield volumes in depression. The analyses in this study involved group comparisons between MDD patients and healthy controls and between TRD patients and healthy controls, as well as regressions for prediction of symptom scores from hippocampal subfield volumes to probe the effects of stress on specific hippocampal domains.

### 4.1. Hippocampal subfield volume differences between patients and controls

Significant volumetric differences between MDD patients and controls were found in CA2/3 in the right hemisphere, with smaller volumes in patients, a pattern which is concordant with the literature on hippocampal subfield changes associated with stress-related disorders (40, 41). Pyramidal neurons in the CA fields are most susceptible to stress and increased cortisol levels (17, 40, 41) which induces neuronal damage, dendritic shrinkage, and reduced astrocyte density, which together may contribute to volume loss (41). This group-level finding persisted in the subset of TRD patients compared to healthy controls, suggesting that hippocampal abnormalities may be further pronounced in patients with resistance to antidepressants. Given the small sample size, however, this finding must be interpreted with caution and validated in a larger population.

### 4.2. Associations between hippocampal subfield volumes and LSC scores

Significant negative associations were found between LSC scores and subfield volumes, specifically left CA1, left CA4/DG, and right entire subiculum. Significant negative associations were also found between LSC and total hippocampal volume in the left and right hemispheres. Investigating associations between LSC score and hippocampal subfields in depression is important to understanding the effects of stress on the hippocampal anatomy, specifically dendritic shrinkage and other neurotoxic effects (24). These findings are concordant with the effects of stress on hippocampal anatomy, specifically affecting the CA fields (21).

### 4.3. Limitations and future directions

A limitation of this study is the small sample size. Despite this, significant differences were found between MDD patients and healthy controls in this dataset, suggesting potential 7T benefits of resolving and identifying subtle structural changes in small subregions including hippocampal subfields. There were also a few subfields with medium to large effect sizes that showed a trend toward an association with MDD, such as the right CA2/3 in Table 2 and right CA4/DG in Table 4. We estimated the *post hoc* power for detecting the difference in right CA2/3 between the MDD patients and the healthy controls to be 43%, with a significance level (alpha) of 0.01 using the Wilcoxon Mann–Whitney test. Caution should be exercised in interpreting the results as low study power can lead to not only decreased likelihood of finding a true association but also overestimation of effect size and low reproducibility. Analysis with a larger sample size or performance of meta-analysis would be necessary to validate these findings (42).

In studying MDD and its relationship to different depression related symptoms, we found that hippocampal subfield CA2/3 was reduced in MDD patients compared to controls as well as in TDR patients versus controls and that reductions in CA1, CA4/DG, and subiculum correlated with lifetime stressors. Leveraging the benefits of ultrahigh field 7T MRI, including superior signal and resolution over clinical field strength MRI, to investigate the hippocampus and its subfields and using a 7T-trained and validated automated segmentation software to that end, can enable overall enhanced detection of imaging markers for MDD and treatment-resistant MDD. Hippocampal subfield volumes may serve as imaging biomarkers for MDD, which may help design more targeted treatments for the disease in the future. Furthermore, we found that there is value in studying MDD and its relationship to different depression related symptoms, especially lifetime stressors, rather than analyzing depression as a binary diagnosis.

## Data availability statement

The raw data supporting the conclusions of this article will be made available by the authors, without undue reservation.

## Ethics statement

The studies involving human participants were reviewed and approved by Icahn School of Medicine at Mount Sinai. The patients/participants provided their written informed consent to participate in this study.

## Author contributions

JA study design, acquisition of data, analysis and interpretation of data, and drafting of manuscript. RF acquisition of data through generating training segmentation data for atlas creation. GV imaging data acquisition and analysis by creating scripts for data extrapolation and for providing key figures. SR recruitment and acquisition of symptom score data, statistical analysis of data including choice of statistical test and results interpretation. K-hH, LX, and PY implementation of automated segmentation software and atlas creation. YJ statistical analysis of data including choice of statistical test and interpretation of results. SB data organization and statistical testing. MK and MS subject recruitment and acquisition of clinical data. H-ML statistical analysis methods chosen and interpretation of results. LF implementation of automated segmentation software. BD study conception and design and analysis and interpretation of data. PH study conception and design and provided training for atlas creation. JM study conception and design, acquisition of data, analysis and interpretation of data. PB contributed substantially to study conception and design, acquisition of data, analysis and interpretation of data, and supervised the study. All authors contributed to the article and approved the submitted version.

## Funding

This research was supported by funds NIH R01 MH109544 and R01HL116953. Icahn School of Medicine Capital Campaign, Translational and Molecular Imaging Institute and Department of Diagnostic, Molecular, and Interventional Radiology.

## Conflict of interest

PB is a named inventor on patents relating to magnetic resonance imaging (MRI) and RF pulse design. The patents have been licensed to GE Healthcare, Siemens AG, and Philips international.

The remaining authors declare that the research was conducted in the absence of any commercial or financial relationships that could be construed as a potential conflict of interest.

## Publisher’s note

All claims expressed in this article are solely those of the authors and do not necessarily represent those of their affiliated organizations, or those of the publisher, the editors and the reviewers. Any product that may be evaluated in this article, or claim that may be made by its manufacturer, is not guaranteed or endorsed by the publisher.

## References

[1] Kessler RC Berglund P Demler O Jin R Koretz D Merikangas KR. The epidemiology of major depressive disorder: results from the National Comorbidity Survey Replication (NCS-R). JAMA. (2003) 289:3095–105. doi: 10.1001/jama.289.23.3095 12813115

[2] Small SA Schobel SA Buxton RB Witter MP Barnes CA. A pathophysiological framework of hippocampal dysfunction in ageing and disease. Nat Rev Neurosci. (2011) 12:585–601. doi: 10.1038/nrn3085, PMID: 21897434 PMC3312472

[3] Krishnan V Nestler EJ. The molecular neurobiology of depression. Nature. (2008) 455:894–902. doi: 10.1038/nature07455, PMID: 18923511 PMC2721780

[4] Hasler G. Pathophysiology of depression: do we have any solid evidence of interest to clinicians? World Psychiatry. (2010) 9:155–61. doi: 10.1002/j.2051-5545.2010.tb00298.x, PMID: 20975857 PMC2950973

[5] Kakeda S Watanabe K Katsuki A Sugimoto K Igata N Ueda I. Relationship between interleukin (IL)-6 and brain morphology in drug-naïve, first-episode major depressive disorder using surface-based morphometry. Sci Rep. (2018) 8:1–9. doi: 10.1038/s41598-018-28300-5 29968776 PMC6030126

[6] Tesen H Watanabe K Okamoto N Ikenouchi A Igata R Konishi Y. Volume of amygdala subregions and clinical manifestations in patients with first-episode, drug-naïve major depression. Front Hum Neurosci. (2022) 15:780884. doi: 10.3389/fnhum.2021.780884, PMID: 35046783 PMC8762364

[7] Murrough JW Charney DS. Is there anything really novel on the antidepressant horizon? Curr Psychiatry Rep. (2012) 14:643–9. doi: 10.1007/s11920-012-0321-8, PMID: 22996298 PMC3662536

[8] Rush AJ Trivedi MH Wisniewski SR Nierenberg AA Stewart JW Warden D. Acute and longer-term outcomes in depressed outpatients requiring one or several treatment steps: a STAR* D report. Am J Psychiatr. (2006) 163:1905–17. doi: 10.1176/ajp.2006.163.11.1905 17074942

[9] Greden JF. The burden of disease for treatment-resistant depression. J Clin Psychiatry. (2001) 62:26–31. 11480881

[10] Sala M Perez J Soloff P Di Nemi SU Caverzasi E Soares JC. Stress and hippocampal abnormalities in psychiatric disorders. Eur Neuropsychopharmacol. (2004) 14:393–405. doi: 10.1016/j.euroneuro.2003.12.005 15336301

[11] Barnes J Ourselin S Fox NC. Clinical application of measurement of hippocampal atrophy in degenerative dementias. Hippocampus. (2009) 19:510–6. doi: 10.1002/hipo.20617, PMID: 19405145

[12] Bremner JD Narayan M Anderson ER Staib LH Miller HL Charney DS. Hippocampal volume reduction in major depression. Am J Psychiatr. (2000) 157:115–8. doi: 10.1176/ajp.157.1.115 10618023

[13] Wise T Radua J Via E Cardoner N Abe O Adams TM. Common and distinct patterns of grey-matter volume alteration in major depression and bipolar disorder: evidence from voxel-based meta-analysis. Mol Psychiatry. (2017) 22:1455–63. doi: 10.1038/mp.2016.72, PMID: 27217146 PMC5622121

[14] Schmaal L Veltman DJ van Erp TG Sämann PG Frodl T Jahanshad N. Subcortical brain alterations in major depressive disorder: findings from the ENIGMA major depressive disorder working group. Mol Psychiatry. (2016) 21:806–12. doi: 10.1038/mp.2015.69, PMID: 26122586 PMC4879183

[15] Duvernoy HM. The human hippocampus: Functional anatomy, vascularization and serial sections with MRI Springer Science & Business Media (2005).

[16] Langston RF Stevenson CH Wilson CL Saunders I Wood ER. The role of hippocampal subregions in memory for stimulus associations. Behav Brain Res. (2010) 215:275–91. doi: 10.1016/j.bbr.2010.07.006, PMID: 20633579

[17] Sapolsky RM. Glucocorticoids and hippocampal atrophy in neuropsychiatric disorders. Arch Gen Psychiatry. (2000) 57:925–35. doi: 10.1001/archpsyc.57.10.925, PMID: 11015810

[18] Sousa N Paula-Barbosa MM Almeida O. Ligand and subfield specificity of corticoid-induced neuronal loss in the rat hippocampal formation. Neuroscience. (1999) 89:1079–87. doi: 10.1016/S0306-4522(98)00311-X, PMID: 10362296

[19] Stockmeier CA Mahajan GJ Konick LC Overholser JC Jurjus GJ Meltzer HY. Cellular changes in the postmortem hippocampus in major depression. Biol Psychiatry. (2004) 56:640–50. doi: 10.1016/j.biopsych.2004.08.022, PMID: 15522247 PMC2929806

[20] Rössler M Zarski R Bohl J Ohm TG. Stage-dependent and sector-specific neuronal loss in hippocampus during Alzheimer's disease. Acta Neuropathol. (2002) 103:363–9. 11904756 10.1007/s00401-001-0475-7

[21] Huang Y Coupland NJ Lebel RM Carter R Seres P Wilman AH. Structural changes in hippocampal subfields in major depressive disorder: a high-field magnetic resonance imaging study. Biol Psychiatry. (2013) 74:62–8. doi: 10.1016/j.biopsych.2013.01.005, PMID: 23419546

[22] Malykhin NV Seres MP Coupland NJ. Structural changes in the hippocampus in major depressive disorder: contributions of disease and treatment. J Psychiatry Neurosci: JPN. (2010) 35:337–43. doi: 10.1503/jpn.100002, PMID: 20731966 PMC2928287

[23] Malykhin N Coupland N. Hippocampal neuroplasticity in major depressive disorder. Neuroscience. (2015) 309:200–13. doi: 10.1016/j.neuroscience.2015.04.047 25934030

[24] Liu W Ge T Leng Y Pan Z Fan J Yang W. The role of neural plasticity in depression: from hippocampus to prefrontal cortex. Neural Plast. (2017) 2017:1–11. doi: 10.1155/2017/6871089, PMID: 28246558 PMC5299163

[25] Yushkevich PA Amaral RS Augustinack JC Bender AR Bernstein JD Boccardi M. Quantitative comparison of 21 protocols for labeling hippocampal subfields and parahippocampal subregions in *in vivo* MRI: towards a harmonized segmentation protocol. NeuroImage. (2015) 111:526–41. doi: 10.1016/j.neuroimage.2015.01.004, PMID: 25596463 PMC4387011

[26] Malykhin N Lebel RM Coupland NJ Wilman AH Carter R. *In vivo* quantification of hippocampal subfields using 4.7 T fast spin echo imaging. NeuroImage. (2010) 49:1224–30. doi: 10.1016/j.neuroimage.2009.09.042, PMID: 19786104

[27] Wisse L Gerritsen L Zwanenburg JJ Kuijf HJ Luijten PR Biessels GJ. Subfields of the hippocampal formation at 7 T MRI: *in vivo* volumetric assessment. NeuroImage. (2012) 61:1043–9. doi: 10.1016/j.neuroimage.2012.03.023, PMID: 22440643

[28] Mueller SG Schuff N Yaffe K Madison C Miller B Weiner MW. Hippocampal atrophy patterns in mild cognitive impairment and Alzheimer's disease. Hum Brain Mapp. (2010) 31:1339–47. doi: 10.1002/hbm.20934, PMID: 20839293 PMC2943433

[29] Brown SS Rutland JW Verma G Feldman RE Alper J Schneider M. Structural MRI at 7T reveals amygdala nuclei and hippocampal subfield volumetric association with major depressive disorder symptom severity. Sci Rep. (2019) 9:1–10. 31308432 10.1038/s41598-019-46687-7 PMC6629636

[30] Katsuki A Watanabe K Nguyen L Otsuka Y Igata R Ikenouchi A. Structural changes in hippocampal subfields in patients with continuous remission of drug-naive major depressive disorder. Int J Mol Sci. (2020) 21:3032. doi: 10.3390/ijms21093032, PMID: 32344826 PMC7246866

[31] Balchandani P Naidich T. Ultra-high-field MR neuroimaging. Am J Neuroradiol. (2015) 36:1204–15. doi: 10.3174/ajnr.A4180, PMID: 25523591 PMC4472608

[32] Pai A Marcuse LV Alper J Delman BN Rutland JW Feldman RE. Detection of hippocampal subfield asymmetry at 7T with automated segmentation in epilepsy patients with Normal clinical strength MRIs. Front Neurol. (2021) 12:682615. doi: 10.3389/fneur.2021.682615, PMID: 34867703 PMC8634833

[33] Wisse LEM Biessels GJ Stegenga BT Kooistra M Van Der Veen PH Zwanenburg JJM. Major depressive episodes over the course of 7 years and hippocampal subfield volumes at 7 tesla MRI: the PREDICT-MR study. J Affect Disord. (2015) 175:1–7. doi: 10.1016/j.jad.2014.12.052, PMID: 25589378

[34] Kraus C Seiger R Pfabigan DM Sladky R Tik M Paul K. Hippocampal subfields in acute and remitted depression—an ultra-high field magnetic resonance imaging study. Int J Neuropsychopharmacol. (2019) 22:513–22. doi: 10.1093/ijnp/pyz030, PMID: 31175352 PMC6672627

[35] Tannous J Godlewska BR Tirumalaraju V Soares JC Cowen PJ Selvaraj S. Stress, inflammation and hippocampal subfields in depression: a 7 tesla MRI study. Transl Psychiatry. (2020) 10:1–7. doi: 10.1038/s41398-020-0759-0 32098947 PMC7042360

[36] Wisse LE Kuijf HJ Honingh AM Wang H Pluta JB Das SR. Automated hippocampal subfield segmentation at 7T MRI. Am J Neuroradiol. (2016) 37:1050–7. doi: 10.3174/ajnr.A4659, PMID: 26846925 PMC4907820

[37] Giuliano A Donatelli G Cosottini M Tosetti M Retico A Fantacci ME. Hippocampal subfields at ultra high field MRI: an overview of segmentation and measurement methods. Hippocampus. (2017) 27:481–94. doi: 10.1002/hipo.22717, PMID: 28188659 PMC5573987

[38] Yushkevich PA Pluta JB Wang H Xie L Ding SL Gertje EC. Automated volumetry and regional thickness analysis of hippocampal subfields and medial temporal cortical structures in mild cognitive impairment. Hum Brain Mapp. (2015) 36:258–87. doi: 10.1002/hbm.22627, PMID: 25181316 PMC4313574

[39] Blanchard G Roquain É. Adaptive false discovery rate control under Independence and dependence. J Mach Learn Res. (2009) 10, 2837–2871.

[40] Travis SG Coupland NJ Hegadoren K Silverstone PH Huang Y Carter R. Effects of cortisol on hippocampal subfields volumes and memory performance in healthy control subjects and patients with major depressive disorder. J Affect Disord. (2016) 201:34–41. doi: 10.1016/j.jad.2016.04.049, PMID: 27162154

[41] Saur L Baptista PPA Bagatini PB Neves LT de Oliveira RM Vaz SP. Experimental post-traumatic stress disorder decreases astrocyte density and changes astrocytic polarity in the CA1 hippocampus of male rats. Neurochem Res. (2016) 41:892–904. doi: 10.1007/s11064-015-1770-3, PMID: 26577396

[42] Button KS Ioannidis J Mokrysz C Nosek BA Flint J Robinson ES. Power failure: why small sample size undermines the reliability of neuroscience. Nat Rev Neurosci. (2013) 14:365–76. doi: 10.1038/nrn3475, PMID: 23571845

